# Pyruvate Dehydrogenase and Tricarboxylic Acid Cycle Enzymes Are Sensitive Targets of Traumatic Brain Injury Induced Metabolic Derangement

**DOI:** 10.3390/ijms20225774

**Published:** 2019-11-16

**Authors:** Giacomo Lazzarino, Angela Maria Amorini, Stefano Signoretti, Giuseppe Musumeci, Giuseppe Lazzarino, Giuseppe Caruso, Francesco Saverio Pastore, Valentina Di Pietro, Barbara Tavazzi, Antonio Belli

**Affiliations:** 1 UniCamillus-Saint Camillus International University of Health Sciences, Via di Sant’Alessandro 8, 00131 Rome, Italy; giacomo.lazzarino@unicamillus.org; 2 Department of Biomedical and Biotechnological Sciences, Division of Medical Biochemistry, University of Catania, Viale A. Doria 6, 95125 Catania, Italy; amorini@unict.it; 3 Division of Emergency-Urgency, UOC of Neurosurgery, S. Eugenio Hospital, Via Filippo Meda 35, 00157 Rome, Italy; stefano.signoretti@aslroma2.it; 4 Department of Biomedical and Biotechnological Sciences, Human Anatomy and Histology Section, School of Medicine, Via S. Sofia 97, 95123 Catania, Italy; g.musumeci@unict.it; 5 Oasi Research Institute–IRCCS, Via Conte Ruggero 73, 94018 Troina (EN), Italy; forgiuseppecaruso@gmail.com; 6 Department of System Medicine, University of Rome Tor Vergata, Via Montpellier 1, 00133 Rome, Italy; pastore@uniroma2.it; 7 Neurotrauma and Ophthalmology Research Group, School of Clinical and Experimental Medicine, College of Medical and Dental Sciences, University of Birmingham, Edgbaston, Birmingham B15 2TT, UK; a.belli@bham.ac.uk; 8 National Institute for Health Research Surgical Reconstruction and Microbiology Research Centre, Queen Elizabeth Hospital, Edgbaston, Birmingham B15 2TT, UK; 9 Institute of Biochemistry and Clinical Biochemistry, Catholic University of Rome, Largo F. Vito 1, 00168 Rome, Italy; 10 Fondazione Policlinico Universitario A. Gemelli IRCCS, Largo A. Gemelli 8, 00168 Rome, Italy

**Keywords:** traumatic brain injury, mitochondrial dysfunction, pyruvate dehydrogenase, tricarboxylic acid cycle, acetyl-CoA, concussion

## Abstract

Using a closed-head impact acceleration model of mild or severe traumatic brain injury (mTBI or sTBI, respectively) in rats, we evaluated the effects of graded head impacts on the gene and protein expressions of pyruvate dehydrogenase (PDH), as well as major enzymes of mitochondrial tricarboxylic acid cycle (TCA). TBI was induced in anaesthetized rats by dropping 450 g from 1 (mTBI) or 2 m height (sTBI). After 6 h, 12 h, 24 h, 48 h, and 120 h gene expressions of enzymes and subunits of PDH. PDH kinases and phosphatases (PDK1-4 and PDP1-2, respectively), citrate synthase (CS), isocitrate dehydrogenase (IDH), oxoglutarate dehydrogenase (OGDH), succinate dehydrogenase (SDH), succinyl-CoA synthase (SUCLG), and malate dehydrogenase (MDH) were determined in whole brain extracts (n = 6 rats at each time for both TBI levels). In the same samples, the high performance liquid chromatographic (HPLC) determination of acetyl-coenzyme A (acetyl-CoA) and free coenzyme A (CoA-SH) was performed. Sham-operated animals (*n* = 6) were used as controls. After mTBI, the results indicated a general transient decrease, followed by significant increases, in PDH and TCA gene expressions. Conversely, permanent PDH and TCA downregulation occurred following sTBI. The inhibitory conditions of PDH (caused by PDP1-2 downregulations and PDK1-4 overexpression) and SDH appeared to operate only after sTBI. This produced almost no change in acetyl-CoA and free CoA-SH following mTBI and a remarkable depletion of both compounds after sTBI. These results again demonstrated temporary or steady mitochondrial malfunctioning, causing minimal or profound modifications to energy-related metabolites, following mTBI or sTBI, respectively. Additionally, PDH and SDH appeared to be highly sensitive to traumatic insults and are deeply involved in mitochondrial-related energy metabolism imbalance.

## 1. Introduction

Mitochondria are the key organelles regulating not only cellular bioenergetic but also redox balance and apoptosis [1]. Undoubtedly, the main mitochondrial function is to provide adequate energy production via ATP. The pathological decrease in this activity negatively affects cellular energy-dependent reactions, which deeply influences other mitochondrial functions and ultimately leads to a decrease in cell survival. The power plant producing the amount of ATP matching the cell energy requirements (ATP synthase or complex V) needs the coordinated action of the four complexes forming the electron transport chain (ETC). ATP synthesis occurs in a stoichiometric ratio, linking the flow of electrons through ETC (*n* = 2 e^−^), the reduction of molecular oxygen (*n* = ½ O_2_) to water (namely, oxygen consumption) and, the amount of protons (*n* = 10 H^+^) transferred into the mitochondrial inner membrane space during the electron flow. The maintenance of the aforementioned stoichiometry allows the synthesis of 2.5-3 or 1.5-2 ATP when the electron donors are, respectively, NADH or FADH_2_. Therefore, ATP production through oxidative phosphorylation (OXPHOS) is strictly dependent on the correct ETC functioning and electroosmotic gradient formation between the intermembrane space and the mitochondrial matrix.

A continuous supply of reducing equivalents, represented by the reduced forms of nicotinic and flavinic coenzymes, is, therefore, needed for proper operation of this system. In healthy mitochondria, the tricarboxylic acid (TCA) cycle (also known as Krebs’ or the citric acid cycle) represents a fundamental metabolic step generating 3 NADH and 1 FADH_2_ at any cycle completion and requires a continuous supply of acetyl-CoA [2]. In glucose metabolism, acetyl-CoA formation occurs via the oxidative decarboxylation of pyruvate, which is catalyzed by the pyruvate dehydrogenase (PDH) complex [3]. This reaction is prodromal to acetyl-CoA entering the TCA cycle and is an additional source of one NADH for each pyruvate transformed by PDH. In cells highly dependent on the metabolism of glucose, such as brain cells, PDH is the only source of acetyl-CoA. The PDH-catalyzed reaction is crucial for the further oxidative metabolism of glucose into the TCA cycle. In these cells, PDH activity guarantees adequate production of NADH and FADH_2_ (via the TCA cycle) and enables the ETC to function, ultimately ensuring ATP generation. Therefore, brain PDH activity and TCA cycle are strictly connected and represent a gauge to assess mitochondrial function energy production [3].

As previously mentioned, the brain may be considered a glucose-dependent organ, at least under physiological conditions [4,5]. The brain meets its energy requirements by completely coupling glucose consumption with oxygen consumption. To ensure this coupling, the following metabolic steps are mandatory: i) pyruvate produced via glycolysis is converted to acetyl-CoA by PDH; ii) the TCA cycle and PDH generate the correct number of NADH and FADH_2_ molecules; iii) ETC efficiently transfers NADH and FADH_2_ electrons to molecular oxygen, while pumping the right number of protons into the mitochondrial inter-membrane space; and iiii) protons are efficiently utilized by ATP-synthase to generate ATP molecules. Under these conditions, glucose consumption almost coincides with glucose oxidation. Dysfunctional mitochondria with reduced oxidative phosphorylating capacities (due to the imbalance in the stoichiometric ratio formed by moles of transferred electrons through ETC:moles of protons pumped by complexes I, III, and IV:moles of oxygen reduced to water:moles of ATP produced by complex V) are a common feature of chronic neurodegenerations. Alzheimer’s disease [6], Parkinson’s disease [7], multiple sclerosis [8], and amyotrophic lateral sclerosis [9] are characterized by an energy deficit causing an increase in apoptosis, as well as oxidative/nitrosative stress due to the excess production of reactive oxygen (ROS) and nitrogen species (RNS). All these phenomena are strictly connected to mitochondrial malfunctioning [10].

There is ample research evidence that traumatic brain injury (TBI), the leading cause of death in the population below 45 years of age in Western countries [11], causes a large number of biochemical and molecular alterations to brain cells, including profound mitochondrial dysfunction [12,13,14]. Following TBI, the derangement of energy metabolism (decrease of ATP and increase in its dephosphorylated products ADP, AMP, adenosine and oxypurines) is well described and has been shown to be proportional to injury severity [15]. In general, metabolic changes associated with mitochondrial functions and energy metabolism have been demonstrated to be transient after mild TBI (mTBI) [16,17] but not after severe TBI (sTBI) [18,19]. As a consequence of altered mitochondrial activity, the onset of oxidative/nitrosative stress [20], with damage to different classes of biomolecules and induction of apoptotic pathways, has also been observed in different models of TBI [21]. As the oxidative metabolism of glucose is significantly compromised, the injured brain undergoes increased glycolytic flux (often termed as hyperglycolysis), even with normal tissue delivery of oxygen [22,23], as a compensatory mechanism attempting to restore adequate ATP for cell energy demands. The duration of this metabolic imbalance and the mechanisms and signals triggering alterations of glucose metabolism are still under investigation [24]. Recently, we demonstrated that following TBI the very early changes of energy metabolites (ATP, ATP/ADP ratio, lactate) are likely the biochemical signals causing later changes in the expression of genes encoding for glycolytic enzymes [25]. Furthermore, these data indicated that sTBI, but not mTBI, induces a true hyperglycolytic state. In sTBI, the gene overexpression of glycolytic enzymes took place during profound mitochondrial malfunctioning, while in mTBI, this overexpression occurred in the recovery phase of mitochondrial functions (normal ATP and ATP/ADP ratio) [19,25].

Employing the same samples used in the aforementioned studies, here we analyzed the time course of changes in the expression of the genes encoding for PDH and its regulatory enzymatic systems (PDH phosphatases and PDH kinases), the genes encoding for the enzymes of the TCA cycle, selected proteins of the TCA cycle, and the concentrations of free CoA-SH and acetyl-CoA in rats receiving mTBI or sTBI.

## 2. Results

### 2.1. Gene Expressions of the PDH Complex and Its Regulatory Enzymes after Graded TBI

**Figure 1:**
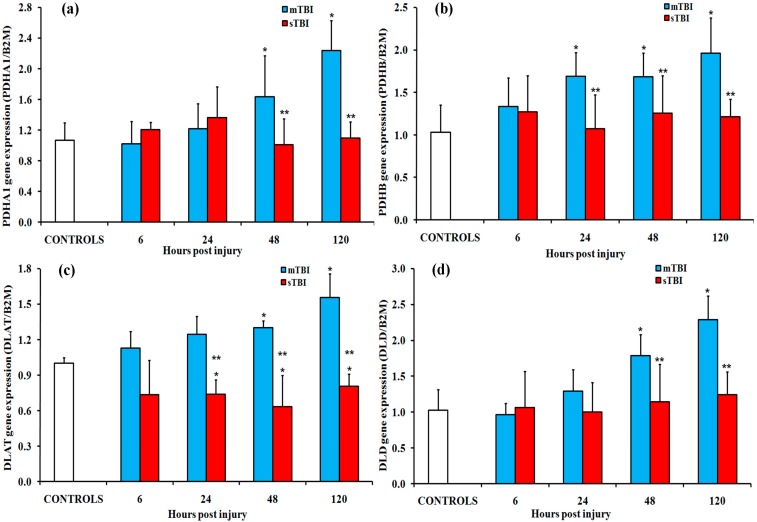
Changes in the expression of genes encoding for the different subunits of the pyruvate dehydrogenase (PDH) complex in brain tissue homogenates of rats receiving traumatic brain injury TBI of different severities (mild (m)TBI or severe (s)TBI), at various times post-injury. (**a**) Pyruvate dehygrogenase E1 alpha (PDHA1) and (**b**) pyruvate dehygrogenase E1 beta (PDHB) genes encoding for the E1 subunits; (**c**) the dihydrolipoamide-S-acetyltransferase (DLAT) gene encoding for the E2 subunit; (**d**) dihydrolipoamide dehydrogenase (DLD), gene encoding for the E3 subunit. Values are the mean of six animals. Standard deviations are represented by vertical bars. Gene expressions were calculated relatively to the expression of the housekeeping gene β-2-microtubulin (B2M). * significantly different from controls, *p* < 0.05. ** significantly different from the corresponding time of mTBI rats, *p* < 0.05.

Changes in the gene expressions of the three enzymes forming the multi-enzymatic PDH complex, namely pyruvate dehydrogenase (E1, PDH; EC 1.2.4.1), dihydrolipoamide-S-acetyltransferase (E2, DLAT; EC 2.3.1.12) and dihydrolipoamide dehydrogenase (E3, DLD; EC 1.8.1.4), in rats receiving mTBI or sTBI at different time points from injury are illustrated in Figure 1.

Significant increases in PDHA1 and PDHB, the two subunits of E1, were selectively measured in mTBI rats at late time points post-injury. At 48 h post injury, PDHA1 and PDHB were, respectively, approximately 1.5 and 2.3 times higher than the controls (*p* < 0.05). At 120 h, they were 1.7 and 2 times higher than the controls (*p* < 0.05). Conversely, during the post-injury observation period, rats experiencing sTBI did not show any changes in their E1 subunits compared to the controls. Concomitantly, E2 (DLAT) and E3 (DLD) in mTBI rats only showed significant increases (1.7 and 1.9 times, respectively) at 120 h (*p* < 0.05 compared to controls). In severely injured rats, significant 30% to 45% decreases were found in DLAT gene expression at 24 h, 48 h, and 120 h post-injury (*p* < 0.05 compared to both controls and the mTBI rats). In these animals, DLD did not show any significant change.

**Figure 2:**
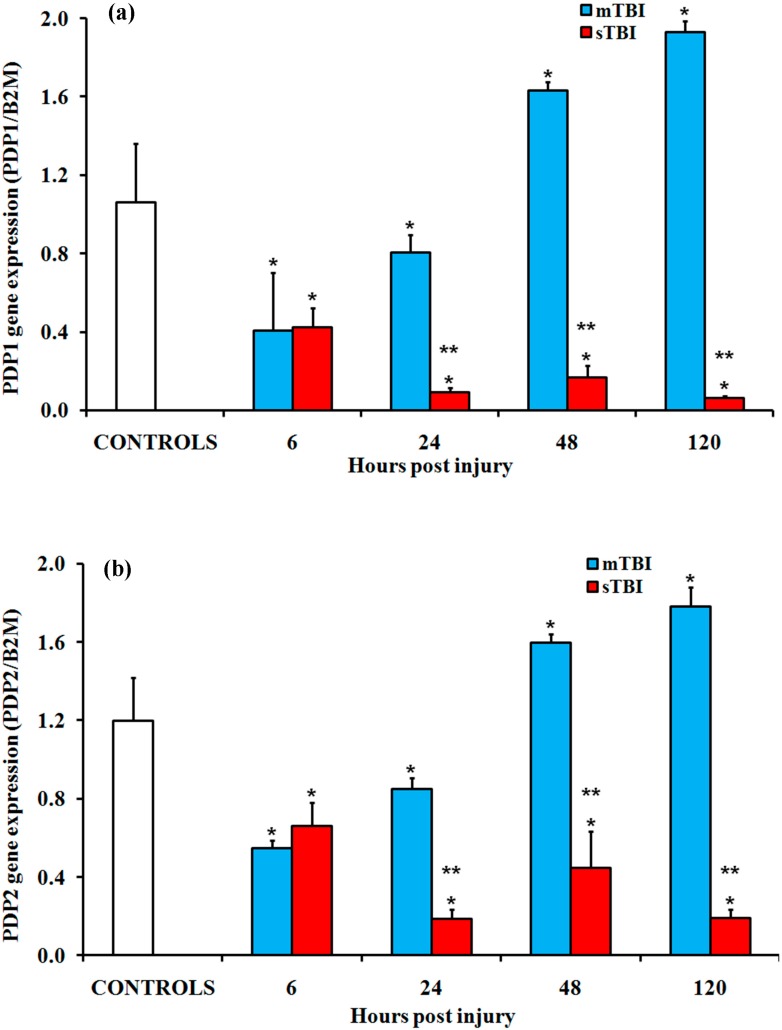
Changes in the expression of genes encoding for the two isoenzymes of pyruvate dehydrogenase phosphatase (PDP) in the brain tissue homogenates of rats receiving a TBI of different severities (mTBI or sTBI) at various times post-injury. (**a**) PDP1, the gene encoding for the isoform 1 of PDP and (**b**) PDP2, the gene encoding for isoform 2 of PDP. Values are the mean of six animals. Standard deviations are represented by vertical bars. Gene expressions were calculated relative to the expression of the housekeeping gene, β-2-microtubulin (B2M). * significantly different from the controls, *p* < 0.05. ** significantly different from the corresponding time of mTBI rats *p* < 0.05.

Since the PDH complex is tightly regulated by dephosphorylating–phosphorylating reactions (respectively, activating and inhibiting the activity of the PDH complex), we also measured the expressions of the genes encoding for the two isoenzymes of pyruvate dehydrogenase phosphatase (PDP1 and PDP2), as well as those encoding for the four isoenzymes of pyruvate dehydrogenase kinase (PDK1, PDK2, PDK3, and PDK4). The data reported in Figure 2 show that both PDP1 and PDP2 in brain extracts of mTBI injured animals at 6 h and 24 h post-injury decreased by approximatively 60% and 30%, respectively (*p* < 0.05 compared to controls). The return to pre-impact values at 48 h and the late increase at 120 h post-injury (1.6 times; *p* < 0.05 compared to controls), were observed after mTBI. Conversely, the PDP1 and PDP2 in sTBI rats were remarkably downregulated at all times post-injury (*p* < 0.05 compared to both the controls and mTBI rats).

**Figure 3:**
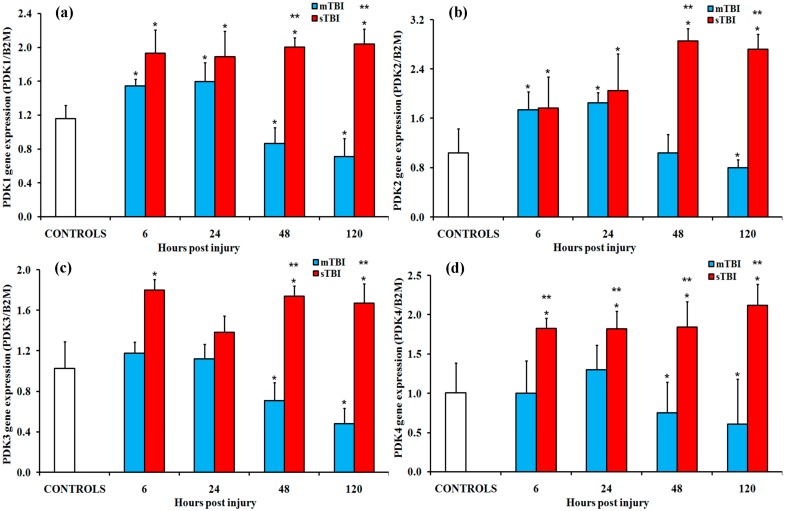
Changes in the expression of genes encoding for the four isoenzymes of pyruvate dehydrogenase kinase (PDK) in brain tissue homogenates of rats receiving a TBI of different severities (mTBI or sTBI) at various times post-injury. (**a**) PDK1, the gene encoding for isoform 1 of PDK; (**b**) PDK2, the gene encoding for isoform 2 of PDK; (**c**) PDK3, the gene encoding for isoform 3 of PDK; and (**d**) PDK4, the gene encoding for isoform 4 of PDK. Values are the mean of six animals. Standard deviations are represented by vertical bars. Gene expressions were calculated relative to the expression of the housekeeping gene β-2-microtubulin (B2M). * significantly different from the controls, *p* < 0.05. ** significantly different from the corresponding time of the mTBI rats, *p* < 0.05.

An opposite trend was recorded in the gene expressions of the four isoenzymes of PDK (Figure 3.). In particular, in mTBI, a modest but significant upregulation of key genes (more evident in the case of PDK1 and PDK4) was mainly found in rats between 48 h and 120 h post- in rats (*p* < 0.05 compared to controls). Rats receiving a sTBI showed a tendency towards a transient upregulation of PDK1, PDK2, and PDK4 at 6 h and 24 h post-injury (*p* < 0.05 compared to controls), with no variations in any of the four PDK subunits at longer times from impact.

### 2.2. Gene and Protein Expressions of the Main Enzymes Regulating the TCA Cycle after Graded TBI

**Figure 4:**
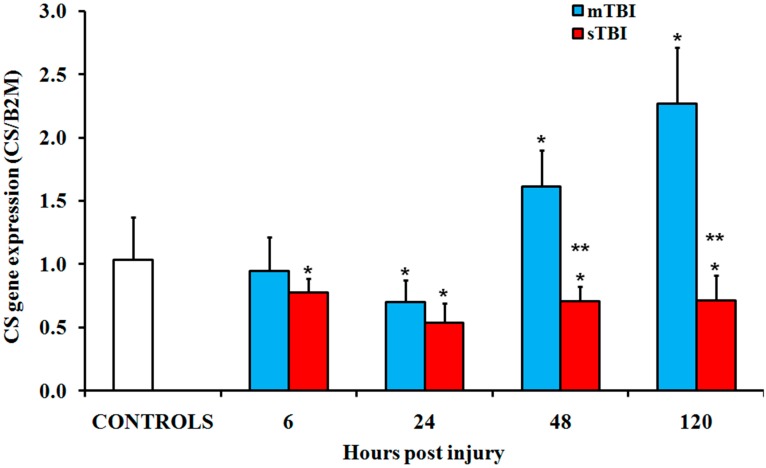
Changes in the expression of the gene encoding for citrate synthase (CS) in the brain tissue homogenates of rats receiving a TBI of different severities (mTBI or sTBI) at various times post-injury. Values are the mean of six animals. Standard deviations are represented by vertical bars. Gene expressions were calculated relatively to the expression of the housekeeping gene β-2-microtubulin (B2M). * significantly different from the controls, *p* < 0.05. ** significantly different from the corresponding time of the mTBI rats *p* < 0.05.

The time course changes in the expressions of the main genes and proteins controlling the TCA cycle following graded TBI were analyzed. In mTBI rats (Figure 4), a transitory downregulation of citrate synthase (CS; EC 2.3.3.1) was detected between 6 h and 24 h after injury, followed by a significant 1.4-fold and 1.8-fold upregulation at 48 h and 120 h, respectively (*p* < 0.05 compared to the controls). Conversely, the gene expression of CS in sTBI animals was downregulated at any time after injury (*p* < 0.05 compared to both the controls and mTBI rats).

**Figure 5:**
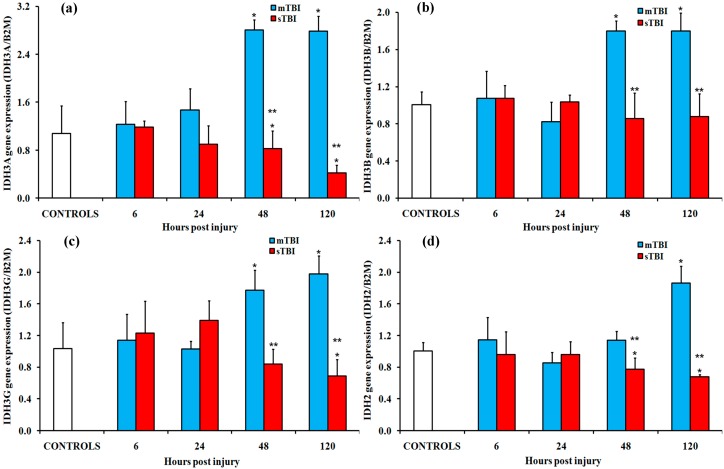
Changes in the expression of genes encoding for the subunits of the isoenzymes of isocitrate dehydrogenase (IDH) in the brain tissue homogenates of rats receiving a TBI of different severities (mTBI or sTBI) at various times post-injury. (**a**) IDH3A, the gene encoding for subunits alfa of the NAD^+^-dependent IDH isoform; (**b**) IDH3B, the gene encoding for subunit beta of the NAD^+^-dependent IDH isoform; (**c**) IDH3G, the gene encoding for subunit gamma of the NAD^+^-dependent IDH isoform; (**d**) IDH2, the gene encoding for the NADP^+^-dependent IDH isoform. Values are the mean of six animals. Standard deviations are represented by vertical bars. Gene expressions were calculated relatively to the expression of the housekeeping gene β-2-microtubulin (B2M). * significantly different from the controls, *p* < 0.05. ** significantly different from the corresponding time of the mTBI rats, *p* < 0.05.

The gene expressions of the subunits of the NAD^+^- and NADP^+^-dependent mitochondrial isocitrate dehydrogenase (IDH) isoforms are shown in Figure 5. The NAD^+^-dependent enzyme (EC 1.1.1.41) is composed of four different subunits (two identical alpha IDH3A, one beta IDH3B, and one gamma IDH3G), while the NADP^+^-dependent isoform (EC 1.1.1.41) is a homodimer (IDH2). Following mTBI, a significant upregulation of each of the aforementioned subunits was recorded in the brain extracts, mainly at 48 h and 120 h from injury (*p* < 0.05 compared to controls). At the same time points, a modest downregulation of all subunits was observed in animals experiencing sTBI (*p* < 0.05 compared to the mTBI rats).

**Figure 6:**
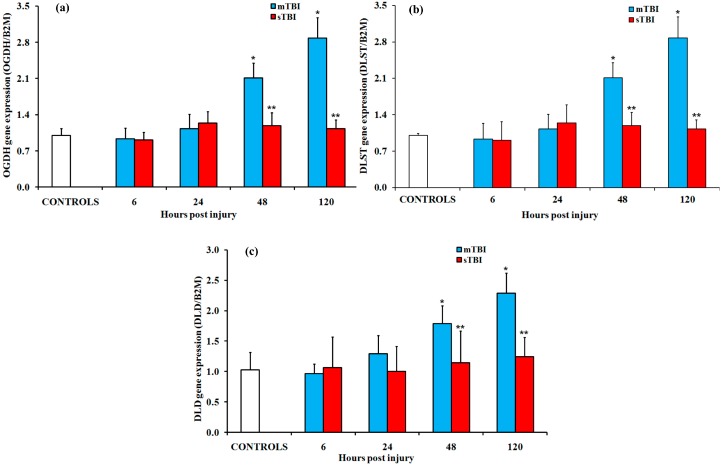
Changes in the expression of genes encoding for the different subunits of the oxoglutarate dehydrogenase (OGDH) complex in brain tissue homogenates of rats receiving a TBI of different severities (mTBI or sTBI) at various times post-injury. (**a**) OGDH, the gene encoding for the E1 OGDH complex subunit; (**b**) DLST, the gene encoding for the E2 subunit dihydrolipoamide-S-succinyltransferase; (**c**) DLD, the gene encoding for the E3 subunit dihydrolipoamide dehydrogenase. Values are the mean of six animals. Standard deviations are represented by vertical bars. Gene expressions were calculated relative to the expression of the housekeeping gene, β-2-microtubulin (B2M). * significantly different from the controls, *p* < 0.05. ** significantly different from the corresponding time of the mTBI rats, *p* < 0.05.

The other multienzymatic complex involved in mitochondrial glucose oxidation, the 2-oxoglutarate dehydrogenase (or α-ketoglutarate dehydrogenase) complex (OGDH complex), is composed of multiple copies of three enzymes: 2-oxoglutarate dehydrogenase (OGDH; EC 1.2.4.2), dihydrolipoamide-S-succinyltransferase (DLST; EC 1.2.4.2), and dihydrolipoamide dehydrogenase (DLD; EC 1.8.1.4). The latter enzyme (DLD) is simultaneously present, with the same primary structure, in the OGDH and PDH multienzymatic complexes, i.e., the expression of the same gene regulates the synthesis of the protein that ensures the correct functioning of both OGDH and PDH. As shown in Figure 6, OGDH, DLST, and DLD showed similar trends. A significant upregulation was detected at 48 h and 120 h in mildly injured rats compared to both the controls and sTBI animals (*p* < 0.05), while no significant changes were measured at any time after sTBI.

**Figure 7:**
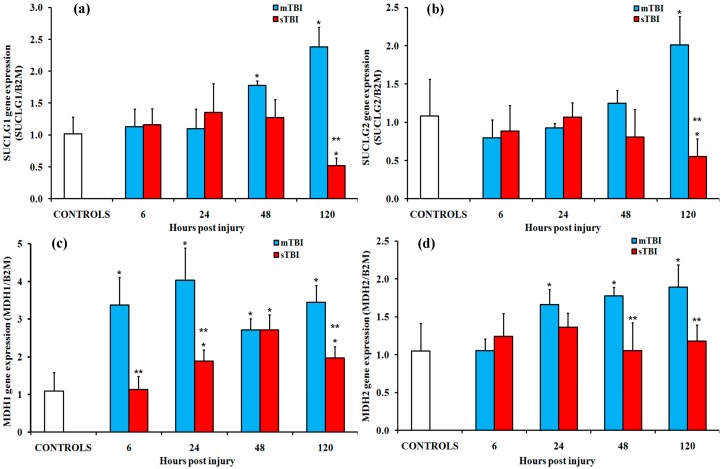
Changes in the expression of the genes encoding for the two subunits of succinyl-CoA ligase (SUCLG1 and 2) and of the cytoplasmic (MDH1) and mitochondrial (MDH2) isoenzymes of malate dehydrogenase (MDH) in the brain tissue homogenates of rats receiving a TBI of different severities (mTBI or sTBI) at various times post-injury. (**a**) SUCLG1, the gene encoding for the α subunit of succinyl-CoA ligase; (**b**) SUCLG2, the gene encoding for the β subunit of succinyl-CoA ligase; (**c**) MDH1, the gene encoding for the cytoplasmic MDH isoform; (**d**) MDH2, the gene encoding for the mitochondrial MDH isoform. Values are the mean of six animals. Standard deviations are represented by vertical bars. Gene expressions were calculated relative to the expression of the housekeeping gene β-2-microtubulin (B2M). * significantly different from the controls, *p* < 0.05. ** significantly different from the corresponding time of the mTBI rats, *p* < 0.05.

The succinyl-CoA synthase (SUCLG or succinyl-CoA ligase, EC 6.2.1.4), is a heterodimer composed of a catalytic α subunit encoded by the SUCLG1 gene and a β subunit encoded by the SUCLG2 gene. Changes in the gene expressions of both subunits are reported in Figure 7A,B. After mTBI, SUCLG1 showed significant 1.4-fold and 1.9-fold increases over the controls at 48 h and 120 h post-injury (*p* < 0.05), while the gene expression of SUCLG2 only significantly increased at 120 h post-injury (*p* < 0.05 compared to the controls). In sTBI, SUCLG1 showed a modest but significant decrease (*p* < 0.05) at 120 h after injury, while SUCLG2 did not differ from the controls at any time post-injury.

The last enzyme of the TCA cycle is malate dehydrogenase (MDH; EC 1.1.1.37). This enzyme catalyzes the reversible oxidation of malate to oxaloacetate and uses NAD^+^ as an electron acceptor. Two main isoforms of MDH exist in eukaryotic cells. The first one (MDH1) is found in the cytoplasm and enables the correct functioning of the malate–aspartate shuttle. This shuttle is essential to transport the electrons of glycolytically generated NADH from the cytoplasm to the mitochondrial matrix and then to the ETC. The other one (MDH2) is found in the mitochondrial matrix and takes part in the TCA cycle. The fold changes of MDH1 and MDH2 are reported in Figure 7C,D. Compared to controls, MDH1 showed a significant upregulation at all time points after mTBI (*p* < 0.05) and at 24 h, 48 h and 120 h after sTBI (*p* < 0.05). MDH2 showed an increase at 24 h, 48 h, and 120 h following mild injury (*p* < 0.05) and no significant changes after sTBI.

**Figure 8:**
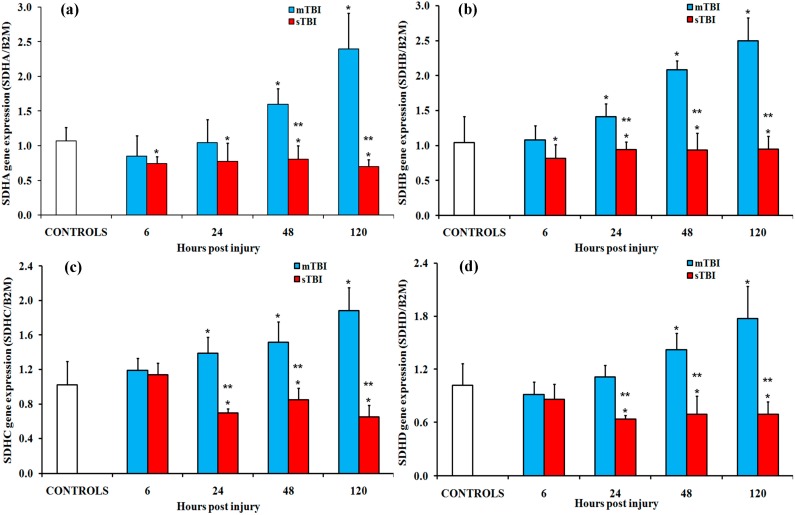
Changes in the expression of genes encoding for the four subunits of succinate dehydrogenase (SDH), or complex II of the electron transport chain (ETC), in brain tissue homogenates of rats receiving a TBI of different severities (mTBI or sTBI) at various times post-injury. (**a**) SDHA, the gene encoding for the A subunit of succinate dehydrogenase; (**b**) SDHB, the gene encoding for the B subunit of succinate dehydrogenase; (**c**) SDHC, the gene encoding for the C subunit of succinate dehydrogenase; (**d**) SDHD, the gene encoding for the D subunit of succinate dehydrogenase. Values are the mean of six animals. Standard deviations are represented by vertical bars. Gene expressions were calculated relative to the expression of the housekeeping gene β-2-microtubulin (B2M). * significantly different from the controls, *p* < 0.05. ** significantly different from the corresponding time of the mTBI rats, *p* < 0.05.

One of the most important enzymes of the Krebs cycle is succinate dehydrogenase (SDH, succinate-ubiquinone oxido-reductase, or complex II; EC 1.3.5.1). This is the only enzyme that participates in both the citric acid cycle and the electron transport chain. Complex II is composed of four subunits: two hydrophilic and two hydrophobic. The first two subunits, the FAD-dependent protein that oxidizes succinate to fumarate (SDHA) and the iron–sulfur protein (SDHB) that transfers electrons from FADH_2_ to coenzyme Q_10_ (ubiquinone), are hydrophilic. The two remaining subunits (SDHC and SDHD) are hydrophobic- membrane-anchored proteins. These are binding sites for oxidized (SDHC) and for reduced coenzyme Q_10_ (SDHD), respectively. As reported in Figure 8, the gene expressions of the four subunits in mildly injured rats were significantly higher than the controls at 48 h and 120 h (*p* < 0.05). In sTBI, a modest downregulation was found for SDHA at 6 h and 120 h, and at 24 h, 48 h, and 120 h for both SDHC and SDHD subunits (*p* < 0.05).

**Figure 9:**
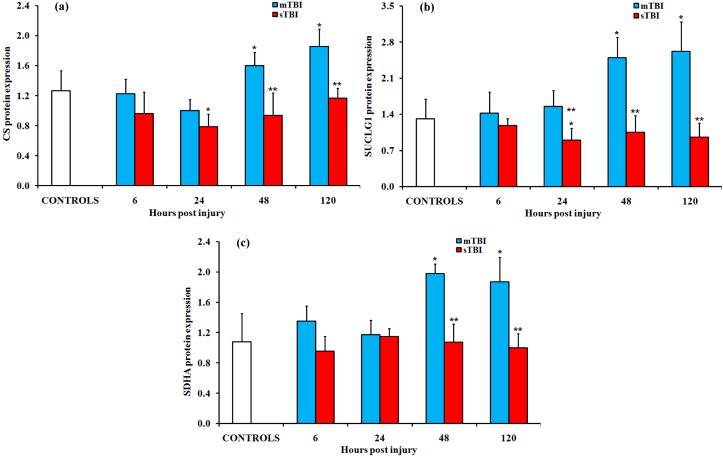
Changes in the protein expression of (**a**) citrate synthase (CS), (**b**) the α subunit of succinyl-CoA ligase (SUCLG1), and (**c**) the A subunit of succinate dehydrogenase (SDHA) in the brain tissue homogenates of rats receiving a TBI of different severities (mTBI or sTBI), at various times post-injury. Values are the mean of six animals. Standard deviations are represented by vertical bars. Protein expressions were measured using proper ELISA kits, as described under Materials and Methods. Values in TBI rats were normalized to the values determined in control sham-operated animals. * significantly different from the controls, *p* < 0.05. ** significantly different from the corresponding time of the mTBI rats, *p* < 0.05.

Among the enzymes of the TCA cycle, we selected CS, SUCLG1, and SDHA to evaluate the protein expressions in the brain homogenates of rats receiving graded TBI. The results (Figure 9) confirmed the data of the gene expression, indicating significant increases in the protein content at later times post mTBI (48 h and 120 h) and relatively rapid significant decreases following sTBI (24 h, 48 h, and 120 h). Of particular relevance were the changes in SUCLG1 and SDHA at 120 h post impact: these changes increased in mildly injured rats and decreased in severely injured animals.

**Figure 10:**
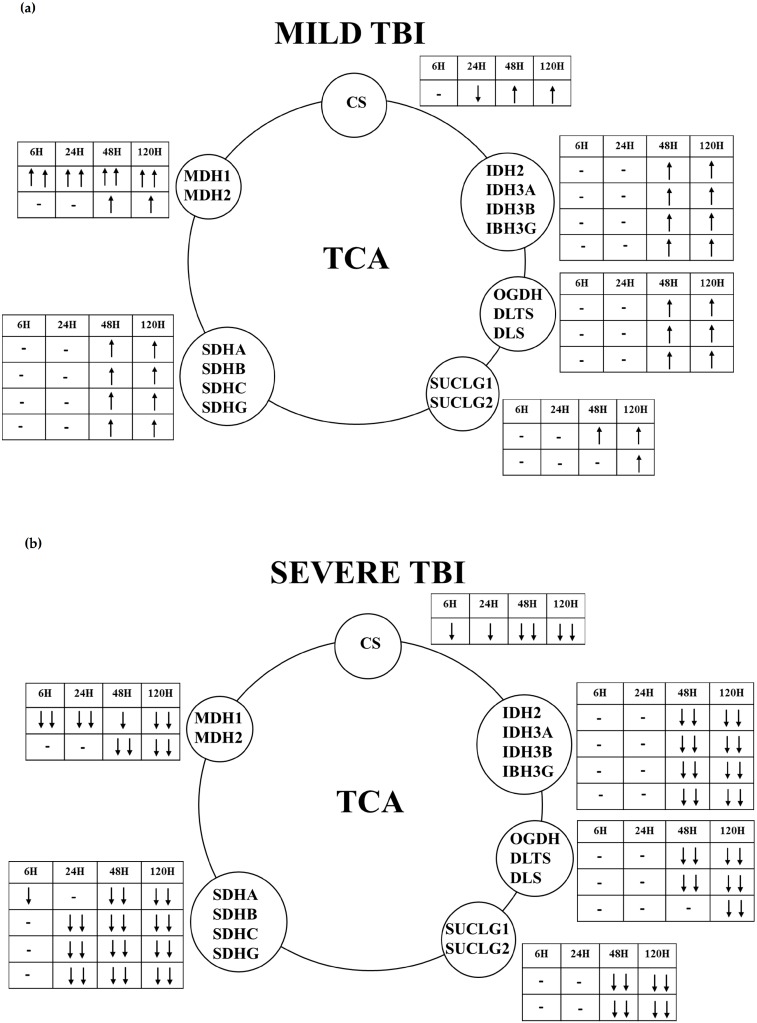
Schematic representations of the changes in the expressions of the genes encoding for the main enzymes of the tricarboxylic acid cycle (TCA) cycle occurring at different times following a mild (**a**) or severe (**b**) TBI.

The schematic summaries of the changes in gene expressions of the enzymes of the TCA cycle following mTBI or sTBI (Figure 10) clearly highlight the dramatic differences occurring between the two levels of head injury, as well as the significant drive to increase energy metabolism late post-injury in the brains of mTBI rats.

### 2.3. CoA-SH and Acetyl-CoAafter Graded TBI

**Figure 11:**
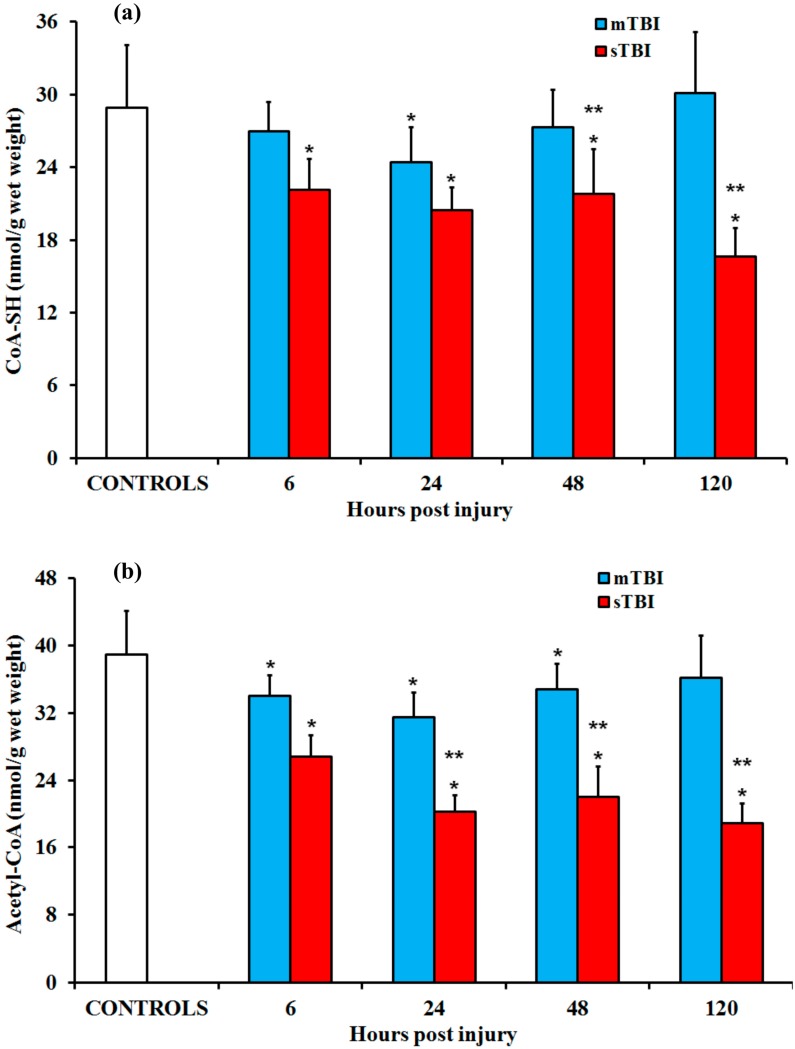
Changes in the concentrations of (**a**) the free coenzyme A (CoA-SH) and (**b**) acetyl-CoA in the brain tissue homogenates of rats receiving TBIs of different severities (an mTBI or sTBI) at various times post-injury. Values are the mean of six animals. Standard deviations are represented by vertical bars. The two compounds were measured by HPLC, as described in Materials and Methods. * significantly different from the controls, *p* < 0.05. ** significantly different from the corresponding time of the mTBI rats, *p* < 0.05.

The changes in the gene expressions of the PDH complex, PDP1-2, and PDK1-4 (Figure 11) were reflected in the CoA-SH and acetyl-CoA concentrations after graded TBI. Rats receiving an mTBI showed no change in CoA-SH and a reversible decrease in acetyl-CoA, while an sTBI led to a concomitant decrease of both CoA forms at any time post injury (*p* < 0.05 compared to the corresponding times of the controls).

The acetyl-CoA/CoA-SH ratio in the controls was 1.35, while at 6 h, 24 h, 48 h, and 120 h post injury, these values were 1.26, 1.29, 1.28, and 1.20 in mTBI, and 1.21, 0.99, 1.01, and 1.14 in sTBI. The sum of the acetyl-CoA + CoA-SH concentrations in the controls was 67.88 nmol/g wet weight (w.w.), while at 6 h, 24 h, 48 h, and 120 h, the post injury values were 60.99, 55.96, 62.16, and 66.29 nmol/g w.w. for mTBI, and 48.93, 40.74, 43.74, and 35.54 nmol/g w.w. for sTBI (*p* < 0.05 compared to both the controls and the corresponding times of the mTBI rats). In other words, a net 48% depletion of acetyl-CoA + CoA-SH occurred under an sTBI, thereby compromising the function of the TCA cycle and inducing a profound derangement of cerebral energy metabolism.

## 3. Discussion

Because of their pivotal role in ensuring cell survival, there is increasing experimental evidence of the direct involvement of mitochondria in the onset and progression of different pathological conditions. TBI has been shown to cause significant changes in various mitochondrial functions, including the transport of electrons through the ETC [26,27], the permeability of the mitochondrial membranes [28], and the mitochondrial quality control system [19,29,30]. These phenomena, besides triggering oxidative/nitrosative stress [20,31] and the intrinsic pathway of apoptosis [32], generate a profound derangement of energy metabolism, leading to inadequate ATP supply for the brain’s energy demands [12,15,16,17,25,33]. Given that the brain mostly utilizes glucose as the fuel for its oxidative metabolism [4,5,34], it is highly relevant to elucidate which steps of glucose oxidation TBI principally affects. The results of the present study indicate that, depending on the severity of the traumatic insult, the PDH complex and enzymes of the TCA cycle are sensitive targets of TBI pathology.

The PDH complex is formed by multiple copies of E1 (PDH), E2 (DLAT), and E3 (DLD). Currently, no data are available in the literature concerning the overall effect of TBI on the genes regulating the expressions of all subunits of E1, E2, and E3. Previous studies have shown that the PDH complex is negatively affected by experimental TBI, either in terms of activity [35], in terms of the expression of the gene encoding for E1 [36], or in terms of oxidative modifications [37]. As the complex is regulated by phosphorylation-dephosphorylation reactions, and catalyzed by PDP (activating PDH) and PDK (inhibiting PDH), we also investigated the influence of TBI on the phosphorylation state of PDH [38,39] and the expression levels of PDP1-2 and PDK 1-4 [35,38].

Using the closed head weight drop model of diffuse TBI in rats, the results reported in the present study provide a full expression pattern of the genes regulating the synthesis of either all the subunits of E1, E2, and E3 or of all the isoforms of PDP (1 and 2) and PDK (1, 2, 3, and 4). We, therefore, demonstrated that different levels of TBI have tremendously different effects on the corresponding gene expressions. In mTBI, an increase in the gene expressions of all subunits of the PDH complex occurred at 48 h and more evidently at 120 h post injury. At the latter time point, an increase of PDP1, inhibition of PDK1, and no changes in the expressions of PDP2 and PDK2-4 were clearly observed. Given that, at early time points post mTBI, PDP1 and PDP2 were significantly downregulated and PDK1, PDK3, and PDK4 were overexpressed, it can be assumed that brain cells adjusted to overall conditions favoring low and high activity of the PDH complex early or late post injury, i.e., when the mitochondria were, respectively, incorrectly or correctly functioning [12,15,16,17,18,19,20,25,28]. Conversely, gene expressions of E1, E2, and E3 in sTBI were either unchanged or slightly decreased (E2), and PDP1-2 was significantly reduced at all times at any time post injury. Together with the significant increase in the PDK1, 2, and 4 isoforms, this strongly suggests that glucose metabolism in a severely injured brain should occur with the PDH complex operating under inhibitory conditions. Hence, the increased energy demand caused by sTBI accelerates glycolysis [25], but the resulting increased flow of pyruvate is not efficiently converted to acetyl-CoA, thus leading to an increase in lactate production [22,25,40,41,42].

Data for the gene and protein expressions of the TCA cycle clearly indicate transient or prolonged mitochondrial dysfunction after mTBI or sTBI, respectively (Figure 10). In a TBI model of the Drosophila melanogaster fly, it was shown that a mild TBI-like injury provoked the transient decrease in the gene expression of IDH and other metabolic genes [43], confirming our previous findings evidencing the gene strategy of neuroprotection adopted by a mildly injured brain [44]. In the present study, we found that gene expressions of CS, IDH isoforms, OGDH, SDH isoforms, and SUCLG isoforms are transiently decreased early post mTBI. Significant increases occurred at later times post injury. The brains of rats experiencing sTBI revealed either no change (IDH3A, IDH3B, IDH3G, OGDH, SUCLG2, SDHB) or a decrease (CS, IDH2, SUCLG1, SDHA, SDHC, SDHD) in the gene expressions of these TCA cycle enzymes. It is worth underlining that in both mTBI and sTBI, the gene expression data were corroborated by the protein expressions of key selected TCA cycle enzymes (CS, SUCLG1 and SDHA). With regard to CS and SDHA, the present results are similar to previous observations obtained in different experimental models of TBI, showing that CS decreased at 24 h post mild-to-moderate TBI [45]. Changes in SDH were also severity-dependent [46,47]. According to the results of protein expressions of CS, SUCLG1, and SDHA (matching those found measuring the expressions of the corresponding genes), it is evident that some time after mTBI the mitochondrial TCA cycle fully recovered. Conversely, the activity of the TCA cycle after sTBI remained at depressed levels compared to pre-impact. This striking difference offers further evidence of short (reversible) or long (permanent) mitochondrial dysfunction depending on the severity of TBI. Bearing in mind that SDH greatly contributes to the direct supply of electrons to the ETC (those of FADH2, gained from the oxidation of succinate), it can also be deduced that their flow through the ETC is only delayed post-mTBI. By contrast, due to the downregulation of the gene and protein expressions of SDH, a decrease in the electron flow through the ETC in mitochondria of sTBI brain cells occurs any time post-sTBI.

Surprisingly, since no data are currently available in the literature, our results are the first to show that SUCLG1, the only enzyme of the TCA cycle that gives a net production of one GTP molecule per molecule of substrate consumed, is negatively affected by sTBI. The decreased gene and protein expressions of SUCLG1 and the overall inhibition of the TCA cycle might indirectly contribute to the well-known phenomenon of glutamate excitotoxicity. This occurs under various states of brain distress, including TBI [18,48,49]. It is conceivable that a decreased rate of the TCA cycle after sTBI may produce an initial increase in the intracellular concentrations of TCA intermediates, particularly keto-acids and more specifically α-ketoglutarate. Instead of being utilized by the TCA cycle, α-ketoglutarate may undergo transamination reaction with aspartate to generate glutamate and oxaloacetate. In sTBI, aspartate has been shown to increase significantly between 6 and 120 h post injury, presumably through the aspartoacylase-meadiated hydrolysis of N-acetylaspartate into acetate + aspartate [16,18]. Therefore, TCA cycle derangement following sTBI might create the perfect conditions to increase glutamate production, thereby contributing to maintaining high glutamate levels in the brain, sustaining excitotoxicity, and impairing the glutamate–glutamine cycle between neurons and astrocytes [18,50].

At the metabolite level, the differential effects of graded TBI on brain metabolism only produced a slight transitory decrease of both free CoA-SH and acetyl-CoA only at a short time after mTBI, whereas there was a consistent 50–60% depletion of both compounds following sTBI. The decrease in acetyl-CoA could certainly be a consequence of the PDH complex inhibition occurring in the sTBI brain. This phenomenon may play a central role in sTBI-induced glucose dysmetabolism, leading to hyperglycolysis and causing an increase in lactate production after sTBI but not after mTBI [22,25,41,42]. It is worth highlighting that severe TBI did not only cause an acetyl-CoA decrease but also provoked a net 48% depletion in the acetyl-CoA + CoA-SH concentration. Under the conditions of energy crisis occurring after sTBI, it is highly plausible that brain cells are not able to perform de novo free CoA-SH re-synthesis starting from its precursors, since this metabolic pathway is characterized by a high energy expenditure (4 ATP moles consumed per mole of CoA-SH produced) [51]. Interestingly, it has been shown that the inborn errors of coenzyme A metabolism are associated with neurodegeneration [52] and that mice in which the degradation rate of free CoA-SH was accelerated by overexpressing peroxisomal hydrolases (the enzymes responsible for free CoA-SH hydrolysis) developed a decrease in motor coordination, as a clear symptom of neurodegeneration [53]. In other words, an imbalance in CoA-SH metabolism may be involved in overt neurologic symptoms frequently present in moderately to severely injured TBI patients.

In conclusion, the data of the present study indicate strikingly differential effects of mTBI and sTBI on the central metabolism involved in energy production. Specifically, the PDH complex appears to suffer profound alterations following sTBI only. Together with the changes occurring to most of the enzymes of the TCA cycle, PDH inhibition may be a critical factor leading to a long term energy crisis. Combined with previous findings obtained in brain samples from the same animals [16,18,19,25,54], it can be concluded that mitochondria are particularly sensitive to the primary insult caused by a mechanical force acting on the brain tissue at the time of impact. However, after an mTBI, the mitochondria may be defined as transiently dormant rather than dysfunctional, adopting a general gene strategy of neuroprotection characterized by a decrease in energy consumption [44]. On the other hand, after sTBI, a plethora of molecular changes (gene and protein expression and concentrations of metabolites) take place for a prolonged period of time. In light of this, pharmacological targets may include the acceleration of the recovery time of mitochondrial functions (in the case of mTBI) or the blockage of molecular events permanently altering cell energy metabolism (in the case of sTBI). According to the results of this and previous animal studies [16,18,19,25,54], it appears that such potential treatments after mTBI may have a better chance to produce benefits if administered relatively late after injury. On the other hand, it looks as though pharmacological treatments after sTBI may have a reasonable chance of success if administered very early post injury. This narrow therapeutic window strongly suggests that new experimental studies should aim to elucidate the timing of drug administration. Furthermore, due the large number of biochemical, metabolic, and molecular changes induced by TBI to brain cells, each representing a potential valid target for pharmacological treatment, it is our strong opinion that studies evaluating the effects of multi-drug administrations should be undertaken. Simultaneously administering drugs interfering with different targets may increase the potential to block multiple mechanisms of TBI-mediated brain metabolic derangement.

## 4. Materials and Methods

### 4.1. Animal and TBI Induction

The experimental protocol was approved by the Ethical Committee of the University of Birmingham, in accordance with international standards and guidelines for animal care. Male Wistar rats of 300–350 g were randomly divided into: 1) rats sham-operated as controls; 2) mild diffuse TBI (mTBI group) rats; and 3) severe diffuse TBI (sTBI group) rats. For the anesthetic mixture, animals received 35 mg/kg b.w. ketamine and 0.25 mg/kg b.w. midazolam by i.p. injection. TBI was induced by dropping a 450 g weight from a 1 m (mTBI) or 2 m height (sTBI), according to the weight drop impact acceleration model [55]. Using this model, mTBI has characteristics similar to those produced by concussive impacts. Rats that suffered from skull fractures, seizures, nasal bleeding, or did not survive the impacts (*n* = 6 in the sTBI group) were discarded and not included in the study. At 6, 12, 24, 48, and 120 h from injury, rats (*n* = 9 for each time point in both groups of injured animals) were again anesthetized and then immediately sacrificed. The sham-operated animals sacrificed 120 h after the initial anesthesia (*n* = 9) were used as the controls.

### 4.2. Tissue Preparation for the Determination of Metabolites, Genes and Protein Expressions

As described in detail elsewhere [12,15,25], an in vivo craniectomy was performed in all animals under anesthesia, and the two hemispheres were freeze-clamped in liquid nitrogen to minimize metabolite loss [16,18,19,25,54]. Total RNA was extracted by homogenizing one hemisphere in Trizol (Invitrogen Life Technologies, Carlsbad, CA, USA), using the Ultra-Turrax homogenizer (Janke Kunkel, Staufen, Ge) at 24,000 rpm/min to produce a final 10% homogenate (w/v). Crude homogenates suitable to measure protein expression were obtained by homogenizing one hemisphere in 15 mM KCl + 1 mM KH_2_PO_4_, pH 7.4, at 24,000 rpm/min for 90 s in the cold, followed by centrifugation at 18,690 × *g* for 15 min at 4 °C. The tissue preparation for the simultaneous HPLC analysis of free CoA-SH and acetyl-CoA was performed on one hemisphere using the organic solvent deproteinization described elsewhere [56]. As previously indicated [16,18,19,25,54], the utilization of these protocols for tissue manipulation, alongside the proper mixing of the different processing of the right and left hemispheres, allowed the simultaneous determination of the gene expressions (three right + three left hemispheres), enzyme activities (three right + three left hemispheres), and quantification of metabolites (three right + three left hemispheres) by using nine animals for each time point.

### 4.3. Determination of Gene Expressions

The transcription to cDNA of RNA extracted from the brain samples and the subsequent real time-quantitative polymerase chain reaction (RT-qPCR) analysis were performed as previously described [19,25,44]. Expressions of the following genes were evaluated: pyruvate dehygrogenase E1, alpha (PDHA1) and beta (PDHB) subunits; dihydrolipoamide-S-acetyltransferase (DLAT); dihydrolipoamide-S-acetyldehydrogenase (DLD); pyruvate dehydrogenase phosphatase, 1 (PDP1) and 2 (PDP2) isoenzymes; pyruvate dehydrogenase kinase, 1 (PDK1), 2 (PDK2), 3 (PDK3) and 4 (PDK4) isoenzymes; citrate synthase (CS); NADP^+^-dependent mitochondrial isocitrate dehydrogenase (IDH2); NAD^+^-dependent mitochondrial isocitrate dehydrogenase, alpha (IDH3A), beta (IDH3B) and gamma (IDH3G) subunits; 2-oxoglutarate dehydrogenase (OGDH); dihydrolipoamide-S-succinyltransferase (DLST); succinyl-CoA synthetase, 1 (SUCLG1) and 2 (SUCLG2) subunits; succinate dehydrogenase (succinate-ubiquinone oxidoreductase or complex I), A (SDHA), B (SDHB), C (SDHC) and D (SDHD) subunits; malate dehydrogenase (cytosolic isoform), (MDH1); and malate dehydrogenase (mitochondrial isoform), (MDH2). The list of primers, designed with the 0.2 version of the Primer3 Input software developed by the Whitehead Institute for Biomedical Research (Cambridge, MA, USA), and using the sequences of *Rattus norvegicus* published by the National Center for Biotechnology Information as templates, is given in Table 1.

For accurate gene expression measurements with RTqPCR, the results were normalized to the housekeeping genes of β-2-microglobulin (B2M, NM_012512) of *Rattus norvegicus*, selected from twelve candidate reference genes using the geNorm Housekeeping Gene Selection Kit (Primer Design Ltd., Southampton, UK). Changes in the transcript abundance of tested genes were calculated using the 2^−ΔΔCT^ method described by Livak and Schmittgen [57].

### 4.4. Determination of Protein Expressions

SUCLG1, CS, and SDHA were quantified using immunoenzymatic ELISA kits (MyBioSource, Inc., San Diego, CA, USA) according to the instructions reported by the manufacturers. Briefly, 100 µL of the standards and supernatants of brain homogenates were incubated in microplate wells pre-coated with antibody specific for each of the three aforementioned proteins. After incubation, biotinylation and a conjugation with streptavidin-horseradish peroxidase plates were incubated for 30 min at 37 °C with 3,3′,5,5′-tetramethylbenzidine. The reactions were stopped by the addition of 50 µL of acidic solution, and the absorbances of the resulting yellow products were measured spectrophotometrically at 450 nm (Molecular Devices, Sunnyvale, CA, USA).

### 4.5. Analysis of Metabolites

The simultaneous separation and quantification of the free Co-SH and acetyl-CoA in deproteinized brain extracts (20 µL) was performed by high performance liquid chromatography (HPLC) according to methods formerly set up in our laboratory [56,58] using a Hypersil C-18, 250 × 4.6 mm, 5 µm particle size column, provided with its own guard column (Thermo Fisher Scientific, Rodano, Milan, Italy). The HPLC apparatus consisted of a SpectraSystem P4000 pump system (Thermo Fisher Scientific, Rodano, Milan, Italy) and a highly-sensitive UV6000LP diode array detector (Thermo Fisher Scientific, Rodano, Milan, Italy), equipped with a 5 cm light path flow cell and set between a 200 and 300 nm wavelength. Assignment and calculations of free Co-SH and acetyl-CoA in the chromatographic runs of brain samples were performed by comparing the retention times, absorption spectra, and area of the peaks (calculated at 260 nm wavelength) of the chromatographic runs of mixtures containing known concentrations of true free Co-SH and acetyl-CoA.

### 4.6. Statistical Analysis

The normal distribution of the data was tested using the Kolmogorov–Smirnov test. The within-group comparison at each time was performed by a one-way analysis of variance (ANOVA). Differences across groups were estimated by a two-way ANOVA for repeated measures. Fisher’s protected least square was used as the post hoc test. Only two-tailed *p*-values of less than 0.05 were considered statistically significant.

## Figures and Tables

**Table 1:** Sequences of the primers used to evaluate the gene expressions (using real-time quantitative PCR) of the subunits and isoforms of the TCA cycle enzymes, as well as of the phosphorylating (PDK1-4) and dephosphorylating (PDP1-2) enzymes regulating the activity of the PDH complex in brain tissue homogenates of control and TBI-injured rats.

Gene Name	Species	Gene Sequence	Forward	Reverse
CS	*Rattus norvegicus*	NM_130755.1	TATGGCATGACGGAGATGAA	CATGAACTTGGGCCTTTCTA
DLAT	*Rattus norvegicus*	NM_031025.1	AGACATCCCCATCAGCAACA	CAACGCTGACATCAACCACA
DLD	*Rattus norvegicus*	NM_199385.2	TGGTTGTTATTGGTGCAGGA	CCCACATGACCCAAAAATTC
DLST	*Rattus norvegicus*	NM_001006981.2	GAGCAGCCTGTAGTAAACGC	TTCATTCTTCCGGGCCTTCT
IDH2	*Rattus norvegicus*	NM_001014161.1	TGGGCCTGCAAGAACTATGA	ACCTTCTCCAGAGTCTGTGC
IDH3A	*Rattus norvegicus*	NM_053638.1	ACACAAATCAGGCCAAGCTG	TGGCCAGGGTACTTATGCAA
IDH3B	*Rattus norvegicus*	NM_053581.1	GATGTGCTTGTGATGCCCAA	ATGGCTGTGGGATTGGCTAT
IDH3G	*Rattus norvegicus*	NM_031551.1	TTGCTAACCCTACTGCCACA	CCACAGCCCGTCCATTAATG
MDH1	*Rattus norvegicus*	NM_001316877.1	GATGGAGCTGCAAGACTGTG	GGTTCCCCACAACAATGACC
MDH2	*Rattus norvegicus*	NM_031151.2	GTTGACTTTCCCCAAGACCA	GTCCACCAGGGAGAAGACAA
OGDH	*Rattus norvegicus*	NM_001017461.1	AACCCTTCCCACTTAGAGGC	TCAGTGGGGTAGGGAGAAGA
PDHA1	*Rattus norvegicus*	NM_001004072.2	TGGAGTTGCAGACATACCGT	AGCTGCTTCCTCGACTTCTT
PDHB	*Rattus norvegicus*	NM_001007620.1	CCCGGTTTGAAAGTGGTCAG	ACTACAGTGATGTGGGTCCC
PDK1	*Rattus norvegicus*	NM_053826.2	TGCACAGTACTTCCAGGGAG	GTCGTCATGTCTTTCGGCTC
PDK2	*Rattus norvegicus*	NM_030872.1	ACCTCAGCCGCATCTCTATC	ACACAGGAGCTTAGCCATGT
PDK3	*Rattus norvegicus*	NM_001106581.1	CTGTGGCATCATTAGCACCC	GGAAGAACCCTGGGACTGAA
PDK4	*Rattus norvegicus*	NM_053551.1	TTGGCTGGTTTTGGTTACGG	CACCAGTCATCAGCCTCAGA
PDP1	*Rattus norvegicus*	NM_001271108.2	GTTCTCTGATGCCATGCCAG	CAGTTCTCCCTTGGCCTACA
PDP2	*Rattus norvegicus*	NM_145091.4	TGGAAACGGGACTAAGCACT	TATGTGCAGGTGAACTCCGT
SDHA	*Rattus norvegicus*	NM_130428.1	GGGGAACATGGAAGAGGACA	AGGAGCTTGCTCTGTCATGT
SDHB	*Rattus norvegicus*	NM_001100539.1	AGACCTTGGCAAAGTCTCGA	ATACTGTTGCTTGCCCTCCT
SDHC	*Rattus norvegicus*	NM_001005534.1	ATTGAATGGGGTCCGACACT	GACAGCACTGCAAGAACCAA
SDHD	*Rattus norvegicus*	NM_198788.2	TTGAATCCCTGCTCTGTGGT	AAGCCCAGCAAAGGTCAAAG
SUCLG2	*Rattus norvegicus*	NM_001100750.1	GGAGTGAGAGTGCAGAGGTT	AACACCTCCTTTCAAACCGC
